# Markers Associated with COVID-19 Susceptibility, Resistance, and Severity

**DOI:** 10.3390/v13010045

**Published:** 2020-12-30

**Authors:** Aisha D. Fakhroo, Asmaa A. Al Thani, Hadi M. Yassine

**Affiliations:** 1 Research and Development Department, Barzan Holdings, Doha 7178, Qatar; asfakhroo@barzanholdings.com; 2 Biomedical Research Center, Qatar University, Doha 2713, Qatar; aaja@qu.edu.qa

**Keywords:** SARS, COVID-19, susceptibility, resistance, pathogenesis

## Abstract

In December 2019, the latest member of the coronavirus family, severe acute respiratory syndrome coronavirus 2 (SARS-CoV-2), emerged in Wuhan, China, leading to the outbreak of an unusual viral pneumonia known as coronavirus disease 2019 (COVID-19). COVID-19 was then declared as a pandemic in March 2020 by the World Health Organization (WHO). The initial mortality rate of COVID-19 declared by WHO was 2%; however, this rate has increased to 3.4% as of 3 March 2020. People of all ages can be infected with SARS-CoV-2, but those aged 60 or above and those with underlying medical conditions are more prone to develop severe symptoms that may lead to death. Patients with severe infection usually experience a hyper pro-inflammatory immune reaction (i.e., cytokine storm) causing acute respiratory distress syndrome (ARDS), which has been shown to be the leading cause of death in COVID-19 patients. However, the factors associated with COVID-19 susceptibility, resistance and severity remain poorly understood. In this review, we thoroughly explore the correlation between various host, viral and environmental markers, and SARS-CoV-2 in terms of susceptibility and severity.

## 1. Introduction

In December 2019, an unusual outbreak of viral pneumonia known as coronavirus disease 2019 (COVID-19) hit Wuhan, China. COVID-19 was then declared as a pandemic in March 2020 by the World Health Organization (WHO) [1]. This novel coronavirus disease is caused by severe acute respiratory syndrome coronavirus 2 (SARS-CoV-2); the newest addition to the coronavirus family. Coronaviruses are positive, single-stranded RNA (ssRNA) viruses that cause diseases in mammals and birds, mainly respiratory and intestinal infections [2]. There are four subgroups of coronaviruses: alpha (α), beta (β), gamma (γ) and delta (δ) [3]. In humans, they cause respiratory tract infections with severity ranging from mild to lethal. Seasonal human coronaviruses (HCoV) such as HCoV-229E, HCoV-NL63, HCoV-OC43, and HCoV-HKU1 contribute to approximately 15–30% of common colds [4]. In addition to the newly emerged SARS-CoV-2, two other highly pathogenic human coronaviruses have emerged in previous years; SARS-CoV (SARS) and MERS-CoV (MERS) [5]. In 2002, the outbreak of SARS started in Guangdong province, China, spreading across 26 countries in the world, affecting about 8096 individuals (9.2% fatality rate) [6]. Ten years later, the first case of MERS emerged in Saudi Arabia, leading to an ongoing endemic in the Middle Eastern region [2,3]. So far, MERS-CoV has affected 2494 individuals, with 34% fatality rate [6]. All three highly pathogenic CoV are of zoonotic origin that belong to the β subgroup [2,3].

The four main protein components of a coronavirus are spike (S), envelope (E), membrane (M), and nucleocapsid (N) proteins [7]. The name “Corona” comes from the crown-like shape of the spike protein on the outer surface of the virus [3]. This S protein is the key to the viral attachment, fusion, and entry to host cell [7]. Compared to the other human coronaviruses, SARS-CoV-2 is closely related to SARS-CoV in terms of sequence and receptor binding. The sequence of SARS-CoV-2′s S protein is approximately 76% and 80% similar to that of SARS-CoV and CoV ZXC21 (i.e., bat-like SARS-CoV), respectively [8]. Further, both SARS-CoV and SARS-CoV-2 bind to the angiotensin converting enzyme 2 (ACE2) receptors, whereas MER-CoV binds to dipeptidyl peptidase 4 (DPP4) [7,8]. ACE2 levels were found to be the highest in the small intestine, testis, kidneys, heart, thyroid, and adipose tissue, followed by medium expression in the lungs, colon, liver, bladder, and adrenal gland [9]. Additionally, enriched ACE2 expression was observed in the nose. According to a recent study, nasal epithelial cells showed the highest ACE2 expression in comparison to the other investigated respiratory cells [10]. ACE2 receptors have been also reported in oral structures, such as the tongue, the floor of the mouth and the saliva [11]. These data suggest that COVID-19 goes beyond being a respiratory disease as it may infect tissues other than the lungs. For example, several studies have reported evidence of SARS-CoV-2 in feces of COVID-19 patients [12], suggesting possible gastrointestinal infections.

SARS-CoV-2 seems to be more transmissible but less pathogenic than other zoonotic-origin CoV. The initial mortality rate of COVID-19 was declared by WHO as 2%; however, this rate has increased to 3.4% as of 3 March 2020 [1]. In fact, different fatality rates have been reported in different countries, with the lowest rate being reported in Singapore (<0.1%) and Qatar (0.2%), and highest in UK (14.8%) [13]. According to several reports, acute respiratory distress syndrome (ARDS) was shown to be the leading cause of death in severely ill COVID-19 patients [14,15,16]. Likewise, ARDS is a common immunological outcome for both SARS and MERS [17], as well as highly pathogenic influenza viruses (H5 and H7) [18]. Patients with severe COVID-19 usually experience a hyper pro-inflammatory immune reaction known as a cytokine storm, which often leads to ARDS, multiple organ failure, and eventually death [5]. Note that people of all ages can be infected with SARS-CoV-2; however, those aged 60 or above and those with underlying medical conditions are more prone to develop severe outcomes. Generally, most COVID-19 cases are actually mild or asymptomatic (80%) [1], meaning that they show mild or no symptoms at all. Based on COVID-19 figures in China, approximately four in five infected individuals are asymptomatic [19]. However, factors related to disease severity or resistance remain poorly understood. There are many extrinsic and intrinsic factors associated with COVID-19 susceptibility, resistance, and severity. These markers include viral, host, genetics, environmental, microbiome, metabolome, blood group, vitamins, and others. In this review, we thoroughly explore the correlation between such markers and SARS-CoV-2 in terms of susceptibility and severity.

## 2. Viral Factors

It has been well established that the human–human transmission rate of SARS-CoV-2 (R0~1.4 to 6.47) is higher than that of both MERS-CoV and SARS-CoV-1 (0.3–1.3 and 2.2–3.7, respectively) [6]. Studies have shown that the spike protein of SARS-CoV-2 harbors a furin-cleavage site (RPAR) that is absent in SARS-CoV-1 and other coronaviruses from the same clade [8,20]. Since this site is cleaved by furin contributing to S protein priming required for viral entry, this site may resemble a “gain of function” mutation, leading to a higher rate of spread in humans [8]. Further, given that furin is abundant in several tissues, it may expand the tissue tropism of SARS-CoV-2 compared to other CoV. This also applies to influenza viruses, where highly pathogenic influenza viruses contain furin-like cleavage sites leading to expansion of tissue tropism [8]. For example, H5N1 hemagglutinin A (HA) cleavage site contains a polybasic insertion (RERRRKKR↓GL), which was shown to be associated with increased virulence of the virus during the Hong Kong 1997 outbreak [21]. In addition, it is known that RNA viruses are continuously evolving, experiencing very high mutation rates that are usually associated with enhanced pathogenicity and virulence [22]. Note that antigenic drift has been observed in other coronaviruses, including SARS-CoV-1 [23]. The mutation rate of SARS-CoV-1 was estimated to be 0.80–2.38 × 10^−3^ substitutions per site per year [24]. On the other hand, the mutation rate of SARS-CoV-2 was estimated to be 1.05–1.26 × 10^−3^ substitutions per site per year, similar to that of some MERS-CoV estimates [25]. Most of the mutations occur in the surface proteins, allowing the virus to escape immune response and enhance pathogenicity [23]. So far, few mutations have been identified in circulating SARS-CoV-2 viruses, but their significance in terms of pathogenicity, transmission, and immune escape has not been identified. A mutation D614G was identified in the spike of SARS-CoV-2; this variant was first identified in Europe in February 2020 and within two months it became the most dominant variant all over the world [23]. Compared to the Wuhan reference sequence, A to G mutation is located at position 23,403 l3 leading to a change in amino acid from aspartic acid to glycine in position 614 [23]. A study has shown that this mutation is frequently found with three other mutations (i.e., transmitted as a haplotype); 241C > T in the 5′ UTR(untranslated region), a silent mutation 303C > T, and 14408C > T mutation that leads to an amino acid change in RNA-dependent RNA polymerase (P323L) [23]. In terms of structure, D614 is not located in the receptor binding domain (RBD) of S protein; rather it is located on the surface of S protein protomer forming a hydrogen bond with neighboring individual S protomers. This hydrogen bonding stabilizes the spike’s mature trimeric form on the virion surface. Thus, the change into glycine would destabilize the hydrogen bonding, possibly altering the interface protomer interactions and glycosylation patterns [23]. Through examining clinical data and SARS-CoV-2 sequences from 999 COVID-19 patients, Korber et al. (2020) identified that D614G was correlated with higher levels of viral RNA in the upper respiratory tract of patients. Additionally, their global tracking data showed that the G614 variant spreads faster than D614, suggesting a higher infectivity of G614 [23]. Based on the pseudotyping in vitro assays, G614 pseudotype virus exhibited a higher infectivity [23]. Interestingly, there was no association between D614G status and the hospitalization status (i.e., clinical severity of the disease). Together, these data suggest D614G is more infectious; however, it does not worsen the clinical outcome. On the contrary, another study has shown a strong correlation between case fatality rates and the G614 variant [26]. Based on their molecular model data, G614 stabilizes the original form of S protein (i.e., unliganded) rather than the activated form, suggesting that this form may be less infective. However, the original form of S protein plays an important role in escaping the immune response. Since this S protein is loosely bound to the receptor and the ACE2 binding site is not exposed, an immune response will not be triggered; hence, shielding the virus from anti-viral-spike antibodies [26]. Thereby, this immunological mechanism is hypothesized to be the cause for higher fatality by G614. It is worth mentioning that higher infectiousness does not always mean higher transmissibility [23], so further studies should look into the impact of G614 thoroughly in vitro and in vivo.

The SARS-CoV-2 RdRp (RNA Dependent RNA Polymerase) (also known as nsp12) has been shown to form a super complex with nsp7 and nsp8 [27]. Additionally, ExoN (Exonuclease) (nsp14) enhances the fidelity of RNA synthesis through proofreading errors made by RdRp [28]. The RdRp mutation (P323L) that is a part of the G614 haplotype has also been reported in another study, which showed that RdRp mutation at position 14480 found in Europe was associated with a higher rate of point mutations compared to that from Asia [28]. Since this mutation is located in the interface domain, possibly regulating the interaction of RdRp with other proteins, including ExoN, nsp8 and nsp7, it is hypothesized that this may contribute to an impaired proofreading ability and in turn a higher rate of mutations [28]. Nevertheless, this mutation’s impact on viral replication is yet to be studied. Another two novel mutations have been reported in nsp6 of SARS-CoV-2 at the amino acidic positions 3691 and 9659 [29]. By analyzing the structure of SARS-CoV-2 protein, including nsp6 mutation, it was shown that this mutation might favor viral infection by playing a role in viral autophagy [29]. Nonetheless, the role of autophagy in SARS-CoV-2 infection needs to be further studied in order to assess the role of the nsp6 mutation.

Collectively, all of these mutations provide potential antiviral therapeutic targets through understanding their role in viral pathogenicity and possible drug resistance. For example, the use of furin inhibitors may inhibit the process of S priming; thus, limiting the viral infection. Regarding the G614 variant, fortunately it showed sensitivity to neutralization when treated with polyclonal convalescent sera, which means antibody therapeutics are still plausible [23]. Note that the G614 status of sera used was unknown, so further experiments should be undertaken to check whether this makes a difference or not. However, higher antibody levels may be required to achieve neutralization since the preliminary results indicate that G614 is more infectious than D614 [23]. RdRp is also an important target for antiviral drugs used in COVID-19, such as remdesivir. Since the P323L mutation in RdRp is located next to a potential docking site, this raises the possibility of a potential role in drug resistance [28]. Therefore, the impact of P323L mutation on RdRp activity should be further assessed.

## 3. Host Factors

### 3.1. Statistics

Studies from Wuhan, China have found that almost 50% of the people with COVID-19 had a co-existing chronic disease (i.e., comorbidity) [30,31]. Other studies from around the globe have also reported severe symptoms of COVID-19 in individuals with underlying medical conditions. In a retrospective study of 1590 COVID-19 subjects in China, the most common comorbidity was hypertension (16.9%), followed by diabetes (8.2%) [32]. Interestingly, immunodeficiency was the lowest, accounting for only 0.2% of the subjects. In this study, it was also shown that more patients with comorbidities including hypertension, cardiovascular diseases, cerebrovascular diseases, diabetes, COPD (Chronic obstructive pulmonary disease), chronic kidney diseases and malignancy progressed to composite end-points (i.e., admission to ICU “Intensive Care Unit”, invasive ventilation or death) compared to those without. Collectively, the data showed that patients with comorbidities experienced worse clinical outcomes compared to those without. In fact, those with two or more comorbidities showed a significant increase in reaching the composite end-points compared to those with one or no comorbidity. In another study of 52 inpatients in China, death was observed in 67% of patients with comorbidities [31]. As mentioned earlier, UK has the highest mortality rate (14.8%). According to a prospective observational cohort study including 20,133 UK COVID-19 inpatients (median age ~73 years), more men were infected than women (60% vs. 40%) and overall mortality corresponded to 26% of patients [33]. The most common comorbidities reported were chronic cardiac disease (31%), uncomplicated diabetes (21%), non-asthmatic chronic pulmonary disease (18%) and chronic kidney disease (16%) [33]. Similar results were observed in another cohort study, where the main factors associated with COVID-19 death were gender (male predominance), older age and associated comorbidities including diabetes, severe asthma, cardiovascular disease and obesity [34]. Therefore, coexisting comorbidities may predispose people to adverse and poor COVID-19 clinical outcomes; this is highly dependent on the type and number of comorbidities. One possible mechanism may be immune dysregulation and inflammation induced by these diseases [35]. However, it is not yet known whether comorbidities contribute to COVID-19 susceptibility.

### 3.2. Diabetes

Diabetes is one of the fastest growing diseases worldwide. Even though it was established that diabetes is prevalent in COVID-19 patients and may lead to severe clinical symptoms, it is not yet verified whether it affects susceptibility to viral infection, and whether these symptoms are a direct outcome of diabetes solely or the renal and cardiovascular comorbidities usually associated with diabetes. The association between diabetes and the virus’ susceptibility/virulence is poorly understood in SARS-CoV-2, but it has been established in other coronaviruses viruses, such as MER-CoV and SARS-CoV. According to a study on MERS, more severe and prolonged lung pathology was observed in type 2 diabetic mice models [36]. This was due to the immune dysregulation including the alteration of important immune mediators such as monocytes/macrophages, CD4+ T cells, Ccl2 and Cxcl10 expression [36]. On the other hand, a study has suggested that SARS-CoV binds to ACE2 receptors in the pancreatic islets, damaging them and eventually leading to acute diabetes [37]. Here, it is the other way around where the viral infection actually causes diabetes. Similar mechanisms may also apply to SARS-CoV-2 infection, considering that both viruses use the same receptor. This phenomenon was also observed with influenza A viruses, where they were shown to be able to infect human pancreatic cells as well as induce pancreatic damage in animal models (in vivo) leading to diabetes [38]. As a result, hyperglycemia may lead to immune imbalance, including impaired monocyte/macrophage functions and pro-inflammatory cytokine productions [39], which may contribute to COVID-19′s severity. In contrast, hypoglycemia was reported at least once in approximately 10% of COVID-19 patients with type 2 diabetes [40]. Hypoglycemia has been associated with pro-inflammatory monocytes’ mobilization and enhanced platelet reactivity [39]. Thus, it is not yet clear whether hyperglycemia or hypoglycemia leads to poor clinical outcomes in COVID-19 patients.

### 3.3. Obesity and Obesogenic Comorbidities

Obesity is a global epidemic that causes a low-grade chronic inflammation, affecting the immune system. The effects include immune response dysfunction, gut microbiome/virome imbalance, pro-inflammatory responses and antiviral immunity reduction [41]. A case control study in Mexico has found that obesity predisposes COVID-19 with the strongest association, followed by diabetes and hypertension [42]. In terms of severity, a study of 30 COVID-19 subjects has reported that patients with a higher BMI (Body Mass Index) experienced more severe symptoms in comparison to those with lower BMI (27.0 ± 2.5 vs. 22.0 ± 21.3) [43]. Excess adiposity caused by obesity may lead to various chronic diseases such as diabetes and hypertension [44]. These obesogenic comorbidities affect the renin–angiotensin system resulting in metabolic imbalance and excess pro-inflammatory response [41]; as a consequence, obese patients with COVID-19 may experience severe symptoms. Obesity is also associated with dysregulation in the production of adipokines (i.e., cytokines secreted by adipose tissue) [44]. For instance, serum amyloid-A acts directly on macrophages, facilitating the increase in inflammatory cytokines secretion, including IL-6 [45], which is an important component of the cytokine storm; commonly observed in severely ill COVID-19 patients. Collectively, these data suggest a possible mechanism in which obesity may influence COVID-19′s clinical severity.

### 3.4. Roles of Host Enzymes

The S glycoprotein is a class I fusion protein that mediates a dual role in the infection process: binding to receptor and fusion with the host membrane. This process is mediated by three main enzymes on the host cells: ACE2, TMPRSS2, and furin. Hence, variants of these enzymes and their expression profiles might play a crucial role in the prognosis of COVID-19 patients.

The proteolytic cleavage of the spike protein at the cleavage site enables its conformational change for virus internalization to host cell [5]. This cleavage is mediated by furin, a protease readily expressed in lung cells. Another readily expressed protease, TMPRSS2, accounts for the spike protein priming as well. On the other hand, studies have shown that ACE2 expression is significantly upregulated in lung tissues of severe COVID-19 patients with comorbidities compared to the control group [46]. ACE2 upregulation is positively correlated with genes involved in histone modifications, such as HAT1, HDAC2 and KDM5B [46]. Hence, it is hypothesized that histone modification (i.e., epigenetic regulation) may contribute to ACE2 upregulation and hence SARS-CoV-2 infection. It is worth mentioning that TMPRSS2 and furin were highly expressed in lungs, but not differentially expressed across lung transcriptomes from COVID-19 patients with comorbidities and the control group. These data suggest that ACE2 may act as a limiting factor for SARS-CoV-2 infection. Therefore, ACE2 upregulation correlates with a higher possibility of severe COVID-19 through mediating SARS-CoV-2 entry into the lung cells.

### 3.5. Medications Associated with Comorbidities

There has been a huge controversy over the effect of the medications ACE inhibitors (ACEIs) and angiotensin receptor blockers (ARBs) on SARS-CoV-2 infection, considering that ACE2 serves as its primary receptor. These medications are usually given to diabetes and hypertension patients. A group argues that these drugs may increase the expression of ACE2; hence, increasing the host’s susceptibility to SARS-CoV-2. ACEIs and ARBS were shown to increases ACE2 expression in the kidneys and heart using animal models, but not in the lungs [47]. On the contrary, some think that they are beneficial since they exhibit immunomodulatory effects by reducing inflammatory responses [39]. As a part of the renin–angiotensin (RAAS) pathway, ACE2 converts Ang II into Ang-(1-7), which exhibits anti-inflammatory activity (i.e., reduces blood pressure and inflammation) [47]. In addition, caution should be taken when administering drugs to diabetic COVID-19 patients considering the hyperglycemic effect of corticosteroids and the hypoglycemic effect of hydroxychloroquine [39].

## 4. Genetics

### 4.1. Overview

Population genetics have been widely associated with susceptibility and resistance to infectious diseases, including viral infections. Geographical variations of COVID-19 have been reported, where the highest rate of infections was observed in Europe (1,544,145) and the lowest in Africa (30,536) [48]. Nevertheless, African Americans correspond to 43% of COVID-19 deaths in the US [49]. A recently published review proposed that the high frequency of p.Ser1103Tyr-SCN5A variant in African Americans makes them susceptible to ventricular arrhythmia (VA) and sudden cardiac death (SCD) induced by COVID-19 (i.e., an intrinsic genetic susceptibility influenced by COVID-19 risk factors including hypoxemia and cytokine storm) [49]. In addition, the Italian-Spanish genome wide association studies (GWAS) identified susceptibility loci at chromosome 3p21.31 with a cluster of several genes associated with respiratory failure in COVID-19 [50]. This risk allele was found at a higher frequency in severe patients requiring oxygen ventilators, suggesting a possible contribution to COVID-19 severity. So, how do the host genetics come into play? It is not yet clear whether it is genetics, social factors and/or a combination of both that contribute to the current geographical variations of COVID-19. Currently, a global database (The COVID-19 Host Genetics Initiative) has been developed to identify genetic factors of COVID-19 in terms of susceptibility and severity (https://www.covid19hg.org/).

### 4.2. ACE2 Gene

The ACE2 gene, located in the X chromosome, is characterized by single nucleotide polymorphisms (SNPs) in the coding region, leading to different allele variants with varying frequencies among different populations [48]. For example, K31R and Y83H protective variants of ACE2 are observed with higher frequencies in Asian populations, whereas those of European descent show a higher frequency of T921 risk variant. In fact, using the S-protein-interacting synthetic mutant map of ACE2, a study has identified natural ACE2 variants that may possibly provide resistance against SARS-CoV-2 infection [51]. These variants include K31R, N33I, H34R, E35K, E37K, D38V, Y50F, N51S, M62V, K68E, F72V, Y83H, G326E, G352V, D355N, Q388L and D509Y. Therefore, the ACE2 polymorphism can affect SARS-CoV-2 susceptibility since the protective ACE2 variants showed diminished binding to the spike protein compared to risk variants. Comorbid conditions associated with COVID-19, such as diabetes and hypertension, are also modulated by ACE2 and the renin–angiotensin system as discussed previously (comorbidity section). Rather than solely being a receptor, ACE2 modulates the downstream inflammatory pathways post-infection [48]. Taken together, ACE2 may play a role in the severity of clinical outcomes in addition to its role in susceptibility to SARS-CoV-2 infection.

**Figure 1:**
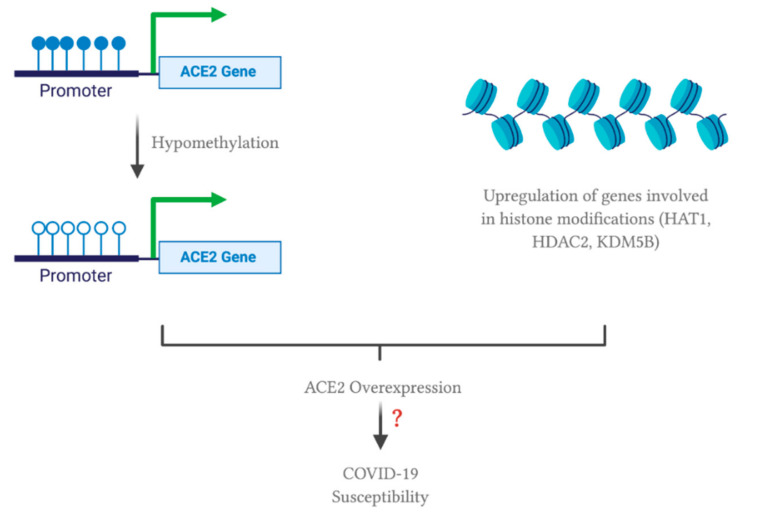
Possible effect of comorbidities on epigenetic regulation of angiotensin converting enzyme 2 (ACE2). All of the figures were created with BioRender.com.

In terms of epigenetic regulation, a study has shown that ACE2 expression can be regulated by DNA methylation in lupus patients [52]. Lupus is an autoimmune disease where the body attacks itself through its hyper-immune system. Hypomethylation of ACE2 was observed in CD4+ T cells, leading to an overexpression of ACE2 in lupus patients compared to the healthy controls. Therefore, oxidative stress induced by COVID-19 in combination with DNA methylation deficiency in lupus patients leads to ACE2 overexpression by inducing hypomethylation at the epigenetic level. In addition, hypomethylation of interferon genes, including NFkB, has been observed. This may correlate with an increase in the cytokine storm. Collectively, these modifications will enhance SARS-CoV-2 entry into lupus patients, increasing their susceptibility to COVID-19. Note that the study did not include data from alveolar epithelial cells in lupus patients. Nevertheless, ACE2 overexpression in immune cells may contribute to SARS-CoV-2 susceptibility and cytokine storm induced organ damage in COVID-19 patients. This phenomenon may also apply to individuals with other comorbidities, explaining why ACE2 is overexpressed in such individuals. As mentioned previously, ACE2 overexpression was correlated with the upregulation of genes involved in histone modifications, such as HAT1, HDAC2 and KDM5B, in individuals with comorbidities [46]. Therefore, a combinational effect of hypomethylation and histone modifications may upregulate ACE2 expression; thus, increasing susceptibility to SARS-CoV-2 infection (Figure 1). Note that there are two isoforms of human ACE2, a full-length transmembrane protein (UniProt ID: Q9BYF1-1, 805 amino acids) and a smaller soluble isoform (UniProt ID: Q9BYF1-2, 555 amino acids) [53]. Since SARS-CoV-2 favors and binds to the membrane-bound ACE2, further studies should look into how epigenetics and the ACE2 polymorphism may account for different isoforms of ACE2, possibly leading to an increased susceptibility or resistance to SARS-CoV-2 infection.

### 4.3. Immune System Related Genes

Regulation of immune related genes may contribute to COVID-19 susceptibility. Human leukocyte antigens (HLA) are proteins encoded by the major histocompatibility complex (MHC) that allow the immune system to differentiate between self and non-self cells. They are characterized by extreme diversity and polymorphisms, accounting for susceptibility against several infectious diseases. In terms of SARS-CoV-2, an in silico study has found that individuals with HLA-B*46:01 variants may be susceptible to COVID-19, whereas HLA-B*15:03 represents a protective variant since it could provide T-cell based immunity [54]. Interestingly, the susceptible allele, HLA-B*46:01, originated in South East Asia; on the other hand, the East Asian gene pool completely lacks the protective allele, HLA-B*15:03 [48]. Therefore, the correlation between HLA variants and COVID-19 needs to be further studied to pinpoint the effect of genetic factors. In relation, a study of sequence analysis has identified 22 variants in the coding regions of some proteases (FURIN, PLG, PRSS1, TMPRSS11a) and innate immune-related genes (MBL2 and OAS1) in a Serbian population [55]. Using in silico analyses, 10 of these variants were predicted to be protein-altering variants, possibly affecting the protein’s function. For example, proteases are involved in proteolytic cleavage of the spike protein, so these variants may provide a “gain of function” mutation enhancing the proteases’ activity. On the other hand, the mutations in innate immune-related genes are hypothesized to be disadvantageous to the host, allowing the virus to escape the immune response. These variants include p.Gly146Ser in FURIN; p.Arg261His and p.Ala494Val in PLG; p.Asn54Lys in PRSS1; p.Arg52Cys, p.Gly54Asp and p.Gly57Glu in MBL2; p.Arg47Gln, p.Ile99Val and p.Arg130His in OAS1 [55]. Additional population genetics studies have shown that seven variants in PLG, TMPRSS11a, MBL2 and OAS1 genes experienced genetic divergence (i.e., different allelic frequencies) among different populations worldwide [55]. It is also interesting to note that cytokine secretion is modulated through genetics and epigenetics regulations. Even though ethnicity has been found to influence the distribution and polymorphisms of cytokines related genes [48], the effect of cytokine gene polymorphisms on SARS-CoV-2 infection has not been studied yet.

## 5. ABO Blood Group

**Figure 2:**
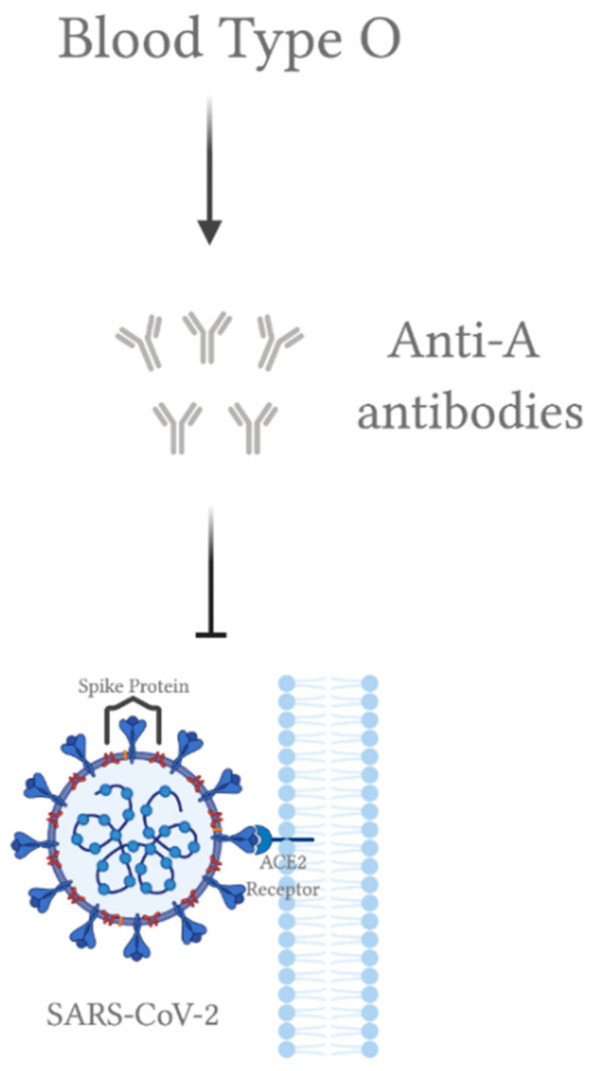
A possible mechanism for severe acute respiratory syndrome coronavirus 2 (SARS-CoV-2) inhibition by blood type O. Anti-A antibodies present in blood type O individuals inhibit the interaction between the S protein of SARS-CoV-2 and ACE2 receptor in host cells. All of the figures were created with BioRender.com.

There are ongoing studies on the relationship between ABO blood group and the host’s susceptibility to SARS-CoV-2 infection. Two independent studies have shown that individuals with blood type A are more susceptible to COVID-19, while blood type O may be protective as it was associated with a decreased risk of infection [56,57]. Based on the results, individuals with blood type A showed a significantly higher rate of COVID-19 infection compared to the control group (57% vs. 38% and 36.90% vs. 27.47%). On the contrary, those with blood type O exhibited a significantly lower COVID-19 infection rate compared to the control group (24.8% vs. 37.2% and 21.92% vs. 30.19%). It is worth mentioning that the Rh factor did not account for any significant difference in neither COVID-19 susceptibility nor its clinical outcomes [56]. Similar results were observed in SARS-CoV-1 infection, where people with blood type O showed lower probability of acquiring SARS in comparison to non-O [56]. A study has used a cellular adhesion model in a hamster to study the possible interaction between ABO natural antibodies and the S protein of SARS-CoV-1 [58]. According to the results, the binding of SARS-CoV-1 S protein to ACE2 in cells was inhibited by either monoclonal or human natural anti-A antibodies; hence, providing protection against viral infection. Therefore, a similar mechanism may apply to SARS-CoV-2, where anti-A antibodies present in individuals with blood type O could provide resistance against SARS-CoV-2 infection through blocking its interaction with ACE2 (Figure 2). Another possible mechanism lies within ABO blood group association with ACE activity. The frequent ABO gene polymorphisms in blood type O carriers (rs8176746, rs8176740, rs495828, rs12683493) were shown to be positively correlated with ACE activity [59], providing possible protection against SARS-CoV-2 infection. If proven, blood type O carriers may be recommended to donate in terms of convalescent plasma therapy for COVID-19. Taken together, ABO blood group correlates with COVID-19 susceptibility, yet it has not been linked to the severity of clinical outcomes.

## 6. Microbiome

The human gut is occupied by 10^4^ microorganisms, including bacteria, archaea, viruses, and fungi. Specifically, the four bacterial *phyla* Actinobacteria, Firmicutes, Proteobacteria, and Bacteroidetes are abundant in the gut of healthy individuals [60]. The balance of such microbiota can be influenced by viral infections in a bidirectional way (i.e., either modulate or be modulated by invading pathogens). In terms of COVID-19, ACE2 is highly expressed in the gastrointestinal tract, and SARS-CoV-2 RNA was detected in stool specimens [12,60]. According to a recent study, 53.42% of COVID-19 hospitalized patients tested positive for SARS-CoV-2 RNA in stool samples, and interestingly, 23.29% tested positive even after showing negative RT-PCR results from nasal/throat swabs [61]. The study further explained the possibility of fecal–oral transmission as they detected SARS-CoV-2 from stool samples. High levels of *Prevotella* were observed in clinical samples of COVID-19 patients; however, it is unclear yet whether it is abundant as a result of viral infection or vice versa [62]. Diarrhea has been also observed in many COVID-19 patients, suggesting a possible cross talk between the lung and the gut. Thereby, it is interesting to investigate the link between gut microbiome and SARS-CoV-2 infection in terms of severity and clinical outcomes in the short and long runs.

Despite the lack of studies on the relationship between gut microbiome and COVID-19, we can speculate possible mechanisms through which the gut microbiome can influence SARS-CoV-2 infection or vice versa, based on the current understanding of gut microbiome physiological functions. The gut microbiome plays an important role in regulating dietary digestion and immunity against pathogens [60]. It also highly impacts cytokine production including type II interferon (interferon-γ), which is critical for antiviral innate and adaptive immune responses [62]. Therefore, the gut’s microbiota may contribute to the hyper-immune response observed in COVID-19 patients (i.e., excessive cytokines production), leading to ARDS and multiple organ failure. Another possible route for SARS-CoV-2 and gut interaction is through the “gut–lung axis”. Similar to the gut, it was shown that the lung also contains a diverse set of microorganisms such as Bacteroidetes, Firmicutes, and Proteobacteria [60]. The gut–lung axis is bidirectional, meaning that there is a crosstalk between the gut and lungs’ microbiota. For example, a gut microbial imbalance can affect the lungs, whereas an inflammation in the lungs can disrupt the gut’s microbiome composition. Similar crosstalk may be observed in COVID-19 patients, influencing its clinical outcomes. Studies have shown that both lung’s and gut’s microbiome are altered in individuals with ARDS [63], which is the leading cause of death in COVID-19.

Healthy and balanced gut microbiota is the key to an optimal immune response against invading pathogens. A study has shown that germfree mice (i.e., with an absence of intestinal microbiota) were more prone to bacterial infection; nevertheless, colonizing them with microbiota restored their ability to respond to the bacterial infection [64]. Thereby, the gut microbiota maintains a balance of immune homeostasis through modulating the cells in between pro- and anti-inflammatory responses [60]. Comorbidities associated with COVID-19, such as diabetes and cardiovascular disorders, usually lead to an imbalance of gut microbiota, known as “gut dysbiosis” [60,62]. As mentioned before, obesity also disrupts the gut microbiome balance [41]. In addition, aging is correlated with a decrease in gut microbiome diversity [65]; this may be the reason for why the elderly are more prone to COVID-19 than children. Together, gut dysbiosis and less diverse microbiome may explain COVID-19 severe clinical outcomes experienced by elderly people with comorbidities. Therefore, a personalized nutritional and diet plan may help restore the beneficial microbiota; hence, enhancing the response against SARS-CoV-2 infection in those with underlying conditions. Probiotics and prebiotics may also be given to help strengthen the immune system.

In addition to the crucial role of gut microbiota in maintaining immune homeostasis, the bacterial residents within the respiratory mucosal surfaces, including the nose, were shown to have immunomodulatory activities. The nasal microbiota prevents pathogen colonization in the nasal cavity through competition for space and nutrients, as well as secreting molecules that can suppress or even kill competing pathogens [66]. The relation between nasal microbiota and some respiratory viral infections has been investigated, such as influenza A virus and rhinovirus. Upon infection with the influenza A virus, the immune system was shown to be modulated by the respiratory mucosa microbiota, specifically CD4 and CD8 T cells production and antibody responses [67]. In this study, a diminished immune response was observed after subjecting the mice to neomycin antibiotics following viral infection, which suggests that the neomycin-sensitive bacteria are crucial for the induction of immune response against influenza A virus. On the contrary, different microbiota clusters in the nasal cavity were associated with varying inflammatory responses (CCL2, CCL20, IL-6, and G-CSF), viral loads, and disease severity in rhinovirus infection [68]. For example, patients with *Pseudomonadaceae*/mixed bacterial clusters were shown to have higher viral loads compared to the other clusters. In addition, patients with *Corynebacterium/Alloiococcus* bacterial clusters experienced less severe symptoms (i.e., lower cold symptoms) compared to the others [68]. Interestingly, the rhinovirus infection did not alter the composition of nasal microbiota [68]. In terms of SARS-CoV-2, a bioinformatics study has proposed that some bacteria of upper respiratory tract microbiota express SARS-CoV-2 spike-binding proteins and these bacteria decline upon aging, suggesting a possible role in COVID-19 infectivity and severity [69]. The members of *Proteobacteria* were shown to have the ability to interact with SARS-CoV-2 spike protein through secretory peptidases and transmembrane/secretory lectins. Interestingly, the *Proteobacteria* population decreases with aging. Thus, this observation may explain why COVID-19 and higher mortality rates are predominant in older individuals. Taken together, studying the composition of respiratory tract microbiota including the nose would give us a better understanding of microbiota–SARS-CoV-2 interactions and their impact on immune responses.

## 7. Metabolome

COVID-19 clinical outcomes are heterogeneous, ranging from mild to lethal symptoms. Approximately 80% of infected individuals exhibit mild symptoms, whereas up to 20% exhibit severe symptoms, specifically respiratory distress requiring inpatient interventions (e.g., ventilations or/and ICU admission) [70]. Therefore, a study developed a system to early identify the cases that will probably develop into severe cases based on the unique molecular changes (i.e., metabolites and proteins) induced by SARS-CoV-2 to avoid mortality [70]. Based on the expression of 22 serum proteins and seven metabolites, it was shown that the severe cases could be identified prior to clinical diagnosis (i.e., even before observing the severe clinical symptoms) with an overall accuracy of 93.5%. The biomarkers used include SAA2 (Serum Amyloid A2), SAA1 (Serum Amyloid A1) and CRP (C-reactive protein). According to the results, the severity of COVID-19 correlated with 204 metabolites found in patient sera (severe vs non-severe COVID-19 patients). For example, there was an increase in glucose, glucuronate, bilirubin degradation products and four bile acid derivatives in sera of severe patients, which will possibly dysregulate the liver’s function leading to hepatic injury. Additionally, major attack complexes (MACs) were upregulated in the severe sera, leading to enhanced cytokine production, and potentially cytokine storms, as observed in COVID-19 severe patients. The impact of comorbidities on the observed metabolomic changes needs to be further studied. Taken together, a targeted therapeutic plan can be applied to severe patients based on the molecular changes of metabolites and proteins observed.

## 8. Vitamins

Vitamin D plays an important role in modulating the adaptive and innate immune system [71]. Specifically, it correlates with lower levels of interleukin-6 (IL-6) [72]; a pro-inflammatory cytokine that regulates the cytokine storm, which leads to ARDS in COVID-19. A study from Ireland has investigated the correlation between vitamin D levels and COVID-19 mortality [73]. By analyzing vitamin D from all European adult population studies since 1999 and comparing it to the mortality rates of COVID-19, this study shows a significant correlation between vitamin D deficiency and COVID-19 mortality rates. For example, lower latitude countries with higher vitamin D deficiency rates (e.g., Spain and Italy) experienced higher infection and death rates compared to Northern latitude countries with lower vitamin D deficiency rates (e.g., Norway, Finland, and Sweden). Therefore, researchers are encouraging the government to apply recommendations for vitamin D supplements since vitamin D may act as a switch from a pro to an anti-inflammatory environment. This is beneficial, especially for older people and those with underlying conditions, such as obesity, diabetes, or hypertension, as their inflammatory response is already at risk.

Other vitamins are also known to have immunomodulatory functions; thus, providing a potential therapy for COVID-19 (i.e., vitamin supplements). Vitamin C has been shown to have important antiviral activity [74], where in vitro inactivation of herpesviruses and paramyxoviruses was achieved upon treatment with ascorbic acid [75]. In terms of immunomodulation, an increase in immune cell activity (i.e., increased expression of CD25 and CD69 of PBMCs (Peripheral blood mononuclear cells) and natural killer (NK) cells) was observed in infected mice (i.e., influenza A virus/H1N1 infection) upon administration of vitamin C [76]. This, in turn, reduced lung inflammation and suppressed the viral lytic cycle. It is also well known that vitamin C is commonly administered to relieve or prevent the symptoms of the common cold and flu [77]. According to a randomized controlled study, daily supplementation of vitamin E and C (800 IU and 1000 mg, respectively) to the HIV-infected population led to a significant decrease in oxidative stress with a possible reduction in HIV viral load [78]. Note that vitamin E poses important antioxidant activities in addition to its anti-inflammatory and immunomodulatory functions [74]. Since the positive effects of these vitamins have been observed in several viral infections, it is worth investigating their therapeutic role in COVID-19, given that the symptoms are quite similar to that of the common cold.

## 9. Immunological Impact of Previous Exposure to Coronaviruses

SARS-CoV-2 is a beta-coronavirus that shares some homology with other coronaviruses, especially SARS-CoV-1. Hence, previous exposure to other coronaviruses may either confer protection or result in enhanced disease illness from the cross-reactivity of B and T cell epitopes and antibodies. Although the SARS-CoV-2 outbreak started in China, Asia accounts for only 8% of global death rates compared to 78% in Europe and North American countries [79]. The previous multiple coronavirus infections that took place in Asian and Middle Eastern countries (i.e., SARS 2003 in China, MERS 2012 in Saudi Arabia) may have built up acquired immunity in such populations leading to an enhanced immune response against SARS-CoV-2 [79]. This may provide a possible explanation as to why lower COVID-19 mortality rates are reported in Asian countries. In fact, several studies have shown that SARS-related antibodies were able to inhibit SARS-CoV-2 pseudovirus entry into host cells (i.e., cross neutralization) [7,20,80]. Additionally, a highly conserved epitope in the receptor binding domain (RBD) of the S protein of SARS-CoV and SARS-CoV-2 has been identified [81]. Researchers have shown that an antibody (CR3022) extracted from a survivor of the SARS epidemic was able to bind to both SARS-CoV and SARS-CoV-2 viruses [81]. The binding sites for CR3022 in both viruses are almost identical (86%) [81]. Although CR3022 was not able to neutralize SARS-Cov-2, this study reveals a vulnerability site in SARS-CoV-2 that can be potentially targeted [81]. The fact that the binding site is highly conserved between SARS-CoV and SARS-CoV-2 opens the door for new possible therapies and vaccines (i.e., other potential shared epitopes). Similarly, in silico study has identified nine antigenic epitopes (presented by MHC class I) conserved among SARS-CoV-2 and other common coronaviruses (OC43, HKU1, 229E, and NL63) that are expected to induce cross-protective immunity [82]. The fact that these coronaviruses are highly prevalent, representing up to 30% of common cold infections every year, suggests that a large proportion of populations worldwide have a pre-existing immunity against SARS-CoV-2 [82]. On the contrary, a study has shown that an anti-SARS-CoV-1 polyclonal antibody (T62) was not able to inhibit SARS-CoV-2 pseudovirus entry into the host cells, meaning that prior exposure to SARS may not provide immunity against COVID-19 [83]. Therefore, it is unclear yet whether previous coronavirus infections provide immunity against COVID-19 or not, which necessitates further investigations.

## 10. Environment

### 10.1. Virus Prevalence in Different Regions and Countries

The effect of the COVID-19 outbreak significantly varies among different countries; ranging from countries that contained the outbreak (e.g., Thailand, Taiwan and Hong Kong) to countries that experienced massive epidemics illustrated by very high infection and mortality rates (e.g., US, UK, Italy, Spain and Iran) [84]. Recently, there has been a rapid increase in COVID-19 cases in Latin America (Southern Hemisphere), specifically in Brazil, followed by Peru (as of April 15) [84]. Even though social distancing was implemented in Peru, the number of cases continued to rise [84]. Therefore, what external/environmental factors come into play? Is it temperature, geographical location, socioeconomics, or medical capacity that contribute to such phenomena? A study has investigated the powerful interaction between demography and population age observed in some countries, such as South Korea and Italy [85]. Upon comparison, both countries have a similar population size but different age structure. Higher mortality rates were observed in Italy, where the overall population age is older than that of South Korea [85]. Therefore, the study encourages governments to make strategical decisions in regard to social distancing based on the age structure of the population [85].

### 10.2. Temperature

Since the beginning of the pandemic, there has been an ongoing controversy over the effect of temperature on SARS-CoV-2 stability. Given the high seasonality of many common coronaviruses (i.e., an increase in December, followed by a peak in January–February and finally a decline in March) [86], it has been suggested that a similar pattern may be observed with SARS-CoV-2. According to a study in Japan, a strong correlation has been observed between lower temperatures and a higher number of COVID-19 cases [87]. In relation, a systematic statistical analysis has proven a negative correlation between the average environmental temperature and the exponential growth rate of COVID-19 cases using data derived from Italian and US regions [88]. Furthermore, the critical temperature (Tc) required for exponential growth elimination in the US was approximately calculated to be 86.1 ± 4.3 °F. A similar negative association was observed in another study, where a one degree Celsius increase in temperature was shown to reduce the *R* value (reproductive number) of COVID-19 by 0.023 and 0.020 in China and US, respectively. Moreover, a one percent increase in relative humidity leads to an *R* value reduction by 0.0078 and 0.0080 in China and US, respectively [89]. It was further expected for the *R* values to decrease by approximately 0.89 in the Northern Hemisphere during summer, assuming the temperature and humidity rise by 30 degrees and 25 percent, respectively. Taken together, these data suggest that the arrival of summer may potentially reduce COVID-19 transmission. On the contrary, a study has shown that warmer weather was not associated with a decline in COVID-19 cases [90]. According to the results, the correlation between ambient temperature and COVID-19 cases was positive in the range below 3 °C and changed to flat above 3 °C [90]. There seem to be opposing opinions regarding the correlation between temperature and COVID-19 transmission; thus, we cannot confirm that higher temperatures will limit COVID-19 transmission yet.

### 10.3. Virus Stability on Surfaces

SARS-CoV-2 tends to differ in stability according to the type of surface it resides on, which may add up to the understanding of viral routes of transmission. Based on the available data, SARS-CoV-2 has a lower stability (i.e., persist for less time) on copper, latex and generally surfaces with lower porosity in comparison to surfaces with high porosity [91]. According to a Lancet report, the infectious virus was undetectable at day four on glass and a banknote, and at day seven on stainless steel and plastic [92]. SARS-CoV-2 maintains its infectivity up to 24 h, 48 h and 72 h on cardboard, stainless steel and plastics, respectively, whereas copper tends to inactivate the virus since SARS-CoV-2 was found to persist up to a maximum of 4 h only on copper surfaces [93]. In terms of disinfections, SARS-CoV-2 is susceptible to many disinfectant agents, including ethanol (62–71%), sodium hypochlorite (0.1%) and hydrogen peroxide (0.5%) [91]. Since SARS-CoV-2 was detected in stool samples of COVID-19 patients and some coronaviruses persist in water for a couple of days, this raises the possibility of fecal–oral transmission [91]. Therefore, further studies should look into the persistence of SARS-CoV-2 in water in order to validate the risk of waterborne transmission of SARS-CoV-2. Additionally, SARS-CoV RNA has been previously detected in human saliva, which raises the possibility of detecting SARS-CoV-2 similarly using non-invasive fluid “saliva” [94].

## 11. Conclusions

**Figure 3:**
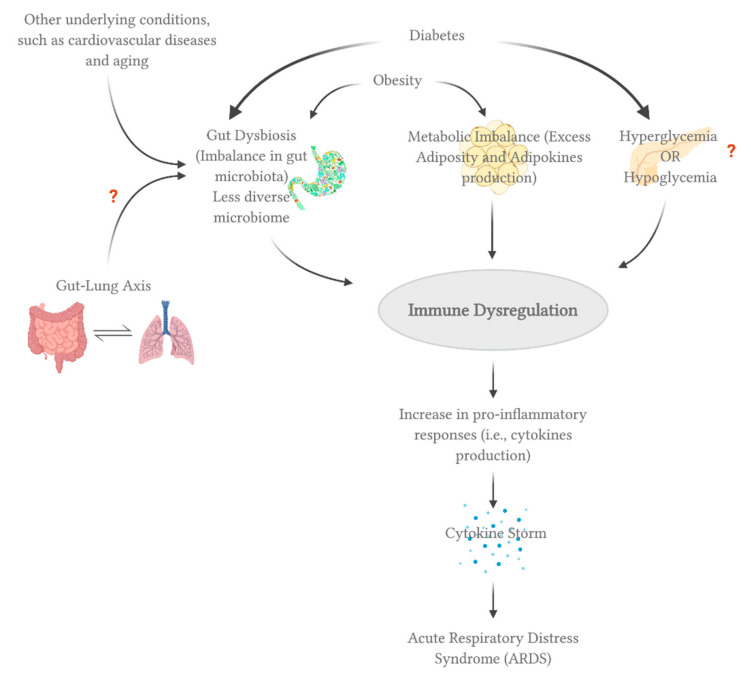
Factors affecting the clinical severity of COVID-19. All of the figures were created with BioRender.com.

**Figure 4:**
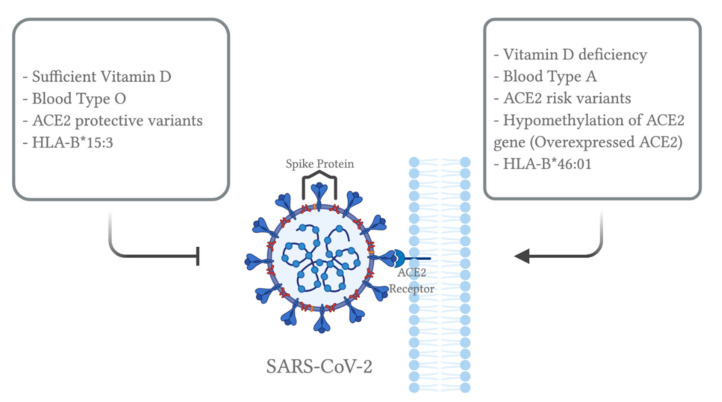
Factors affecting susceptibility to SARS-CoV-2 infection. All of the figures were created with BioRender.com.

In conclusion, understanding the individual and combinational role of host, viral and environmental factors in SARS-CoV-2 infection will provide a better insight into the potential high risk groups of people for COVID-19 in terms of both susceptibility and severity. Figure 3 describes how some of these factors, such as comorbidities, microbiome, and metabolome, come together to possibly produce a combinational effect leading to immune dysregulation and increased severity of COVID-19. Furthermore, the risk vs. susceptible markers against SARS-CoV-2 infection are summarized in Figure 4, specifically emphasizing the role of ABO blood group, vitamin D and intrinsic genetic factors. Therefore, investigating each factor and possible interactions will give us a better understanding of potential antiviral therapy.

## Data Availability

Not applicable.
